# Illness and tretment representation in onological patients undergoing chemotherapy: relationship with subjective well-being

**DOI:** 10.1192/j.eurpsy.2022.379

**Published:** 2022-09-01

**Authors:** E. Rasskazova, S. Kirsanova

**Affiliations:** 1 Mental Health Research Center, Medical Psychology, Moscow, Russian Federation; 2 Moscow State University, Clinical Psychology, Moscow, Russian Federation

**Keywords:** psycho-oncology, chemotherapy, illness representation

## Abstract

**Introduction:**

The knowledge, expectations, fears that a patient has about the oncological disease and treatment can affect the quality of life of patients (Colagiuri et al., 2013; Whitford, 2012).

**Objectives:**

The aim was to reveal the relationship between well-being of patients with cancer undergoing chemotherapy and their illness and treatment representation.

**Methods:**

110 patients undergoing chemotherapy in Medsi Clinical Hospital filled Chemotherapy Attitudes Questionnaire (Zinchenko et al., 2020), Life Satisfaction Scale (Diener et al., 1985), Scale of Positive and Negative Experience (Diener et al., 2009), Quality of Life Questionary C30 (Aaronson N. K. et al., 1994), Illness Perception Questionnaire (Moss-Morris et al., 2002), Self-Regulation Questionnaire in the Rehabilitation Process (Kovyazina M. et al, 2019), Hospital Anxiety and Depression Scale (Zigmond, Snaith, 1983).

**Results:**

Correlation analysis revealed that patients with severe difficulties in physical functioning had a lower level of life satisfaction (R = -0.23, p <.05) and quality of life (R = -0.35, p <.001), perceived disease as long-term (R = 0.34; p <0.001), cyclical (R = 0.33; p <0.001) and carrying significant negative consequences for life (R = 0.55; p <0.001), also these patients were characterized by anxiety about health during treatment (R = 0.37; p <0.001).

Perception of illness duration, personal control, emotional representations, self-efficacy, confidence in the effectiveness of treatment can predict the level of satisfaction with life of cancer patients undergoing chemotherapy (R^2^ increased from 0.05 to 0.37, p<0.001).

**Conclusions:**

Health anxiety, illness duration, personal control, self-efficacy could be targets for interventions in patients undergoing chemotherapy.

**Disclosure:**

No significant relationships.

